# Epidemiology of monkeypox notifications in the state of Minas Gerais, Brazil

**DOI:** 10.1590/0034-7167-2022-0598

**Published:** 2023-10-09

**Authors:** Paula Luciana Gonçalves Pereira, Edmundo Rinolino Magalhães Flores, Thales Philipe Rodrigues da Silva, Ana Paula Vieira Faria, Elice Eliane Nobre Ribeiro, Ana Paula Sayuri Sato, Larissa Pereira Gomes, Fernanda Penido Matozinhos

**Affiliations:** I Secretaria Estadual de Saúde de Minas Gerais. Belo Horizonte, Minas Gerais, Brazil; II Universidade Federal de Minas Gerais, Belo Horizonte, Minas Gerais, Brazil; III Universidade de São Paulo. São Paulo, São Paulo. Brazil

**Keywords:** Epidemiology, Monkeypox, Public Health, Disease Notification, Communicable Diseases, Epidemiología, Viruela del Mono, Salud Pública, Notificación de Enfermedades, Enfermedades Transmisibles, Epidemiologia, Varíola dos Macacos, Saúde Pública, Notificação de Doenças, Doenças Transmissíveis

## Abstract

**Objectives:**

to describe the epidemiological profile of suspected, confirmed, and probable cases of monkeypox in the state of Minas Gerais, Brazil.

**Methods:**

a descriptive, retrospective study of reported suspected, confirmed, and probable cases of monkeypox infection in the state of Minas Gerais, Brazil. The study period was from the first notification, on June 11, to September 7, 2022.

**Results:**

a total of 759 suspected, confirmed, and probable cases of monkeypox infection were reported, with 35.44% suspected, 53.75% confirmed, and 10.81% probable cases, respectively. As for the coexisting diseases within confirmed cases, 38.79% were related to people living with human immunodeficiency virus, and 13.74% had some active sexually transmitted infection. Regarding the evolution of confirmed cases, 47.43% were cured.

**Conclusions:**

the results contribute to greater knowledge and control of the infection by allowing better disease management and care offered in health services.

## RESUMO

**Objetivos:**

descrever o perfil epidemiológico dos casos suspeitos, confirmados e prováveis por *monkeypox* no estado de Minas Gerais, Brasil.

**Métodos:**

estudo descritivo, retrospectivo, com os casos notificados suspeitos, confirmados e prováveis de infecção pelo *monkeypox* no estado de Minas Gerais, Brasil. O período do estudo foi desde a primeira notificação, em 11 de junho, até 7 de setembro de 2022.

**Resultados:**

foram notificados 759 casos suspeitos, confirmados e prováveis de infecção pelo *monkeypox*, sendo, respectivamente, 35,44% suspeitos, 53,75% confirmados e 10,81% prováveis. Quanto às doenças coexistentes nos casos confirmados, 38,79% referiam-se a pessoas vivendo com vírus da imunodeficiência humana, e 13,74% possuíam alguma infecção sexualmente transmissível ativa. Sobre a evolução dos casos confirmados, 47,43% evoluíram para a cura.

**Conclusões:**

os resultados contribuem para maior conhecimento e controle da infecção, de modo a auxiliar no melhor gerenciamento da doença e cuidado ofertados nos serviços de saúde.

## RESUMEN

**Objetivos:**

describir perfil epidemiológico de casos sospechosos, confirmados y probables por *viruela símica* en Minas Gerais, Brasil.

**Métodos:**

estudio descriptivo, retrospectivo, con casos notificados sospechosos, confirmados y probables de infección por *viruela símica* en el estado de Minas Gerais, Brasil. El período del estudio fue desde la primera notificación, en 11 de junio, hasta 7 de septiembre de 2022.

**Resultados:**

fueron notificados 759 casos sospechosos, confirmados y probables de infección por *viruela símica*, siendo, respectivamente, 35,44% sospechosos, 53,75% confirmados y 10,81% probables. Cuanto a las enfermedades coexistentes en los casos confirmados, 38,79% referidos a personas viviendo con virus de la inmunodeficiencia humana, y 13,74% poseían alguna infección sexualmente transmisible activa. Sobre la evolución de casos confirmados, 47,43% evolucionaron para la cura.

**Conclusiones:**

los resultados contribuyen para mayor conocimiento y control de la infección, auxiliando en la mejor gestión de la enfermedad y cuidado ofrecidos en los servicios de salud.

## INTRODUCTION

The recent outbreak of the disease caused by the monkeypox virus has provoked a worldwide public health concern. It is a zoonosis caused by a virus of the *orthopoxviruses* genus of the *Poxviridae* family^(1)^. The first time the disease was reported in humans was in 1970, in a child in the Democratic Republic of Congo, and the outbreaks were initially contained in the African continent, affecting mainly poor populations^(2)^.

Recently, however, new cases have begun to be reported outside of the endemic regions (West Central Africa): on May 7, 2022, the United Kingdom reported one case in its region; and on May 23, 2022, the World Health Organization (WHO) reported 93 cases in 12 countries^(3)^. The reduction in population immunity caused by low vaccination coverage may have set the stage for the reemergence of the monkeypox virus^(2)^.

As of August 30, 2022, 47,751 cases and 15 deaths have been reported worldwide^(4)^. In Brazil, the first case was reported on May 31, 2022, and by August 30, 4,216 cases had been reported, most of them in the Southeast Region^(5)^; among these cases, one death was reported. Most cases were observed in males with homosexual orientation^(6)^.

Disease transmission occurs through respiratory droplets, coughing, and sneezing. The droplets do not travel more than a few meters, and prolonged contact is usually necessary for transmission to occur. In addition, other known methods of transmission are contact with the viral lesion and body fluids, indirect contact with infected clothing or materials, and possible sexual contact^(7-8)^.

After contact with the virus (nasopharyngeal, intradermal, or oropharyngeal), it replicates, spreading to local lymph nodes. The incubation period lasts from seven to 14 days, with a limit of 21 days^(8)^. The main clinical features reported are viral prodrome, fever, myalgias, and back pain, in addition to exanthema and maculopapular rash^(9)^. On July 23, 2022, the WHO declared the current monkeypox outbreak as a Public Health Emergency of International Importance due to the speed with which the virus has spread, especially in non-endemic countries, and especially due to the lack of relevant evidence and information on the subject and changes in the biological aspects of the virus^(10)^.

With the worsening of the outbreak in several regions and the scarcity of evidence on the management and characteristics of the disease, it should be better investigated.

## OBJECTIVES

To describe the epidemiological profile of suspected, confirmed, and probable cases of monkeypox in the state of Minas Gerais (MG), Brazil.

## METHODS

### Ethical aspects

Due to the nature of this study, which relies on open access data, it was not necessary to submit the project to the Research Ethics Committee according to resolution 466/2012 of the National Health Council.

### Study design, location, and period

This is a descriptive, retrospective study, of the reported suspected, confirmed, and probable cases of monkeypox infection in the state of Minas Gerais. The study period was from the first notification, on June 11, to September 7, 2022. Data collection took place on September 22, 2022, using the data available in the RedCap system. It should be noted that, currently, all new cases of monkeypox are reported within 24 hours by health professionals from public or private services via the *Sistema de Informação de Agravos de Notificação* [Notifiable Diseases Information System], called “e-SUS” (https://esussinan.saude.gov.br/login), of the Ministry of Health. The STROBE (Strengthening the reporting of observational studies in epidemiology) instrument was used to guide the study’s methodology^(11)^.

The state of Minas Gerais is composed of 853 municipalities, distributed in a territorial area of 586,528 km^2^, with a population of 21,168,791 inhabitants in 2019, configuring itself as the second most populous state in Brazil. The Minas Gerais Health Regionalization Master Plan proposes an administrative division of the territory in 14 health macro-regions, with the objective of organizing and planning health care in their respective areas of coverage.

### Sample, inclusion, and exclusion criteria

For this research, all notified suspected, confirmed, and probable cases of monkeypox disease were evaluated. A case is considered suspected when:

the individual, of any age, presents sudden onset of mucosal lesion and/or acute rash suggestive of monkeypox, be it single or multiple, located anywhere in the body (including genital/perianal, oral region) and/or proctitis (e.g., anorectal pain, bleeding), and/or penile edema, and may be associated with other signs and symptoms^(3,12)^.

Regarding probable cases, these are defined as:

a case that meets the definition of a suspected case, which presents one or more of the following criteria, with inconclusive or non-performed laboratory investigation of monkeypox, and whose diagnosis of monkeypox cannot be ruled out only by clinical laboratory confirmation of another diagnosis:

(a) Close and prolonged exposure without respiratory protection, or direct physical contact, including sexual contact, with multiple and/or unknown partners within 21 days prior to the onset of signs and symptoms; and/or
b) Prolonged close exposure without respiratory protection, or history of close physical contact, including sexual contact, with a probable or confirmed case of monkeypox within 21 days prior to the onset of signs and symptoms; and/or
c) Contact with contaminated materials, such as bed and bath linens or commonly used utensils, belonging to those with a probable or confirmed case of monkeypox within 21 days prior to the onset of signs and symptoms; and/or
(d) healthcare workers without proper use of personal protective equipment (PPE) with a history of contact with a probable or confirmed case of monkeypox in the 21 days prior to the onset of signs and symptoms^(3,12)^.

Finally, confirmed cases are defined as: “suspected case with a “Positive/Detectable” monkeypox virus (MPXV) laboratory result by molecular diagnosis (Real-Time PCR and/or Sequencing)” ^(3,12)^.

### Study protocol

In this study we described the demographic characteristics related to sexual activity, type of contact with suspected case, symptoms, comorbidities, lesion characteristics, and case evolution. We used notified data available in a public online platform, and data collection was carried out by researchers trained for this purpose.

### Analysis of results and statistics

The data obtained were registered in a Microsoft Office Excel^®^ 2010 spreadsheet. They were then analyzed using the Statistical Software for Professional (Stata) statistical package, version 16.0, and presented using absolute and relative frequency. It is worth saying that The number of patients in some categories may vary due to lack of information at the source. The results were described and presented in tables.

## RESULTS

Within the study period, 759 suspected, confirmed, and probable cases of monkeypox infection were reported in the state of Minas Gerais, with 35.44% suspected, 53.75% confirmed, and 10.81% probable.

Among the confirmed cases, most were male at birth (98.53%), with a median age of 33 years (IQR: 28-37), white race/color (46.22%), cisgender (76.63%), homosexual (71.28%), and had multiple partners (50.47%). Regarding the suspected cases, most were brown (37.17%), heterosexual (51.84%), and did not report having multiple partners (62.64%) (Table 1).

**Table 1:** Description of reported cases, Minas Gerais, Brazil, 2022

Variables	Confirmed cases	Suspected cases	Probable cases
	n (%)	n (%)	n (%)
Sex at birth			
Female	6 (1.47)	105 (39.03)	33 (40.24)
Male	402 (98.53)	164 (60.97)	49 (59.76)
Age^*^	33 (28-37)	27 (21-31)	29 (19-41)
Race/color			
Yellow	4 (1.16)	1 (0.52)	1 (1.39)
White	159 (46.22)	74 (36.11)	26 (36.11)
Brown	109 (31.69)	71 (37.17)	25 (34.72)
Black	40 (11.63)	24 (12.57)	12 (16.67)
Data not available	32 (9.30)	21 (10.99)	8 (11.11)
Gender Identity			
Cisgendered	282 (76.63)	83 (34.02)	30 (40.54)
Trans man	6 (1.63)	1 (0.41)	-
Trans woman	-	2 (0.82)	-
Non-binary	8 (2.17)	2 (0.82)	3 (4.05)
Transgender	6 (1.63)	1 (0.41)	-
Data not available	67 (18.21)	97 (39.75)	19 (25.68)
Sexual orientation			
Bisexual	31 (7.81)	8 (3.27)	4 (5.00)
Heterosexual	34 (8.56)	127 (51.84)	44 (55.00)
Homosexual	283 (71.28)	28 (11.43)	16 (20.00)
Other	4 (1.01)	8 (3.27)	-
Data not available	35 (8.82)	70 (28.57)	13 (16.25)
Men who have sex with men			
No	33 (8.92)	31 (49.21)	31 (49.21)
Yes	305 (82.43)	20 (31.75)	20 (31.75)
Data not available	32 (8.65)	12 (19.05)	12 (19.05)
Other sexual conducts			
Sexual relations with men	143 (74.09)	66 (54.55)	27 (69.23)
Sexual relations with men and women	27 (13.99)	7 (5.79)	2 (5.13)
Sexual relations with women	23 (11.92)	48 (39.67)	10 (25.64)
Multiple partners			
No	64 (30.19)	109 (62.64)	24 (52.17)
Yes	107 (50.47)	18 (10.34)	14 (30.43)
Data not available	41 (19.34)	8 (17.39)	8 (17.39)

* *Median and interquartile range*

*Note: The number of patients in some categories may vary due to lack of information at the source.*

*Source: RedCap.*

Regarding contact with a suspected case, most cases reported as confirmed, suspected, or probable had no close exposure (56.78%, 75%, and 36.59%, respectively). However, the confirmed cases had direct contact (64.87%), including sexual contact (14.79%), with a suspect case (Table 2).

**Table 2:** Description of reported cases and contact with a suspected case, Minas Gerais, Brazil, 2022

Variables	Confirmed cases	Suspected cases	Probable cases
	n (%)	n (%)	n (%)
Contact with a suspected case			
Close exposure			
Yes	66 (16.88)	8 (3.39)	29 (35.37)
No	222 (56.78)	177 (75.00)	30 (36.59)
Data not available	103 (26.34)	51 (21.61)	23 (28.05)
Direct contact - including sexual			
Yes	253 (64.87)	24 (10.13)	52 (63.41)
No	97 (24.87)	167 (70.46)	24 (29.27)
Data not available	40 (10.26)	46 (19.41)	6 (7.32)
Intimate contact - including sexual			
Yes	38 (14.79)	5 (2.24)	13 (20.63)
No	148 (57.59)	175 (78.48)	31 (49.21)
Data not available	71 (27.63)	43 (19.28)	19 (30.16)

*Note: The number of patients in some categories may vary due to lack of information at the source.*

*Source: RedCap.*

The most prevalent symptoms were sudden onset of fever and skin rash, for suspected, confirmed, and probable cases (Table 3).

**Table 3:** Description of the presence of symptoms, Minas Gerais, Brazil, 2022

Variables	Confirmed cases	Suspected cases	Probable cases
	n (%)	n (%)	n (%)
Sudden onset of fever			
Yes	215 (61.17)	70 (26.67)	24 (30.50)
Adenomegaly			
Yes	208 (50.98)	35 (13.01)	28 (34.15)
Acute skin rash			
Yes	330 (80.88)	221 (82.16)	63 (76.83)
Headache			
Yes	167 (40.93)	72 (26.77)	23 (28.05)
Back pain			
Yes	72 (17.65)	27 (10.04)	9 (10.98)
Asthenia			
Yes	145 (35.54)	46 (17.10)	23 (28.05)
Muscle pain			
Yes	130 (31.86)	45 (16.73)	20 (24.39)
Conjunctivitis			
Yes	8 (1.96)	4 (1.49)	3 (3.66)
Nausea/Vomiting			
Yes	49 (12.01)	22 (8.18)	12 (14.63)
Photosensitivity			
Yes	11 (2.70)	7 (2.60)	1 (1.22)
Sweat and chills			
Yes	114 (27.94)	29 (10.78)	13 (15.85)
Sore throat			
Yes	111 (27.21)	41 (15.24)	21 (25.61)
Signs of hemorrhage			
Yes	8 (1.96)	2 (0.74)	2 (2.44)
Arthralgia			
Yes	38 (9.31)	8 (2.97)	6 (7.32)
Coughing			
Yes	24 (5.88)	28 (10.41)	7 (8.54)
Widespread lymphadenopathy			
Yes	10 (2.45)	4 (1.49)	2 (2.44)
Localized lymphadenopathy			
Yes	115 (28.19)	22 (8.18)	14 (17.07)
Mucosal lesions			
Yes	20 (4.90)	10 (3.72)	3 (3.66)
Genital/perianal lesions			
Yes	179 (43.87)	50 (18.59)	25 (30.49)
Oral lesions			
Yes	36 (8.82)	16 (5.95)	3 (3.66)
Penile edema			
Yes	36 (8.82)	6 (2.23)	2 (2.44)
Proctitis			
Yes	39 (9.56)	7 (2.60)	6 (7.32)
Other symptoms			
Yes	49 (12.01)	38 (14.13)	15 (18.29)

*Note: The number of patients in some categories may vary due to lack of information at the source.*

*Source: RedCap.*

Concerning the characteristics of the lesions, most cases presented multiple lesions in the genital region (Table 4).

**Table 4:** Description of the characteristics of lesions, Minas Gerais, Brazil, 2022

Variables	Confirmed cases	Suspected cases	Probable cases
	n (%)	n (%)	n (%)
Lesion(s)			
Multiple	214 (92.24)	185 (93.43)	50 (96.15)
Single	18 (7.76)	13 (6.57)	2 (3.85)
Facial lesions			
Yes	57 (13.97)	54 (20.07)	11 (13.41)
Trunk lesions			
Yes	100 (24.51)	122 (45.35)	23 (28.05)
Lower limbs lesions			
Yes	64 (15.69)	100 (37.17)	22 (26.83)
Upper limbs lesions			
Yes	85 (20.83)	115 (42.75)	26 (31.71)
Genital lesions			
Yes	147 (36.03)	51 (18.96)	21 (25.61)
Anal lesions			
Yes	71 (17.40)	15 (5.58)	8 (9.76)
Oral lesions			
Yes	32 (7.84)	13 (4.83)	4 (4.88)
Palm lesions			
Yes	18 (4.41)	21 (7.81)	4 (4.88)
Bottom of foot lesions			
Yes	8 (1.96)	11 (4.09)	4 (4.88)
Other lesions			
Yes	18 (4.41)	24 (8.92)	5 (6.10)

*Note: The number of patients in some categories may vary due to lack of information at the source.*

*Source: RedCap.*

Finally, it was observed that most patients were not immunosuppressed. However, it is noteworthy that in the confirmed cases, 38.79% were people living with HIV, 13.74% had some active sexually transmitted infection (STI), the most prevalent being syphilis (51.85%). As for the evolution of the confirmed cases, one patient died because of the infection, and 47.43% of them were cured (Table 5).

**Table 5:** Description of reported cases and presence of sexually transmitted infection, Minas Gerais, Brazil, 2022

Variables	Confirmed cases	Suspected cases	Probable cases
	n (%)	n (%)	n (%)
Immunosuppression			
No	253 (64.21)	19 (7.85)	62 (77.50)
Yes - Cause unknown	-	1 (0.41)	-
Yes - Due to medication	2 (0.51)	2 (0.83)	-
Yes - Due to some disease	110 (27.92)	12 (4.96)	14 (17.50)
Data not available	29 (7.36)	19 (7.85)	4 (5.00)
People living with HIV			
No	212 (53.40)	190 (77.87)	56 (70.00)
Yes	154 (38.79)	16 (6.56)	15 (18.75)
Data not available	31 (7.81)	38 (15.57)	9 (11.25)
Active STI			
No	254 (64.63)	193 (78.46)	54 (67.50)
Yes	54 (13.74)	5 (2.03)	7 (8.75)
Data not available	85 (21.63)	48 (19.51)	19 (23.75)
Which STI?			
Gonorrhea	9 (16.67)	-	1 (16.67)
HPV	2 (3.70)	-	-
Genital herpes	2 (3.70)	-	-
Lymphogranuloma Venereum	2 (3.70)	-	-
Syphilis	28 (51.85)	5 (83.33)	5 (83.33)
Others	7 (12.96)	-	-
Hospitalization			
Yes	24 (6.25)	5 (2.19)	3 (3.70)
No	339 (88.28)	208 (91.23)	76 (93.83)
Data not available	21 (4.86)	12 (5.26)	2 ( (2.47)
Outcome			
Cured	157 (47.43)	16 (7.84)	1 (1.39)
Death by monkeypox	1 (0.30)	-	-
Death due to something else	1 (0.30)	-	-
Data not available	173 (52.27)	188 (92.16)	71 (98.61)

*Note: The number of patients in some categories may vary due to lack of information at the source.*

*Source: RedCap.*

## DISCUSSION

This study described the epidemiological and clinical characteristics of 759 infected, suspected, or probable cases of monkeypox reported in the second most populous state of Brazil. The data can contribute to a better understanding of the epidemiological profile of the disease. We can observe cases of community transmission, unrelated to travel to countries where there are endemic cases^(13)^, and that possibly occurred due to person-to-person contact or by possible sexual transmission, a means of transmission unknown until now.

Most of the individuals in this study are male, homosexuals, or men who have sex with men. These observations are like those of a study conducted in four regions (Europe, Americas, Western Pacific, and Eastern Mediterranean), which found that sexual activity between men or bisexual men was the most likely form of transmission^(14)^. This finding may be justified by the presence of primary lesions on the oral, anal, and genital mucosa, which may be the source of disease transmission^(14-15)^. Studies have demonstrated that there is a potential role of sexual contact as a promoter of transmission^(16)^.

In addition, a higher prevalence of infected individuals with multiple partners is observed. It is noteworthy that 38.79% of infected individuals are people living with HIV and 13.74% reported having some active STI, with syphilis being the most prevalent (51.85% of cases). Such groups are at higher risk for STIs, and there are cases of monkeypox virus infection among individuals living with HIV who are on antiretroviral treatment. However, HIV/MPXV coinfection needs to be further investigated, despite some studies showing the potential for infection of the disease in HIV-immunosuppressed patients^(17-18)^.

About the clinical manifestations, sudden onset of fever, adenomegaly, acute rash, and headache were predominant in infected individuals, but none of them presented severe symptoms; and in 88.28% of the confirmed cases, the individuals did not require hospital admission. In some of the cases, lesions were observed in the genital and perianal region. These clinical manifestations have also been reported in other studies^(7,19)^. In the present study, fever was reported in 61% of the confirmed cases, a result similar to that found in a study carried out in the United Kingdom, which reported fever in 57% of cases^(20)^. In addition, it was observed that 80.88% of the confirmed cases presented skin rash, similar to that described in a study carried out in 16 countries, which reported skin lesions in 95% of individuals^(14)^.

Lesions in the genital and perianal regions occurred in 43.87% of infected individuals and 18.59% in suspected cases, and studies have also reported the presence of lesions in these regions observed in the current outbreak of the disease^(21)^. Such characteristic diverges from the previously known forms of the disease, in which lesions start on the face, hands, and feet, further reinforcing the previously stated new possibility of transmission, through sexual contact.

Finally, concerning the characteristics of the lesions, 92.24% of infected individuals presented multiple lesions, 24.51% had lesions on the trunk, and 36.03% showed genital lesions, characteristics also reported in other studies^(14,21)^. Still, it is important to highlight that regarding the evolution of the confirmed cases, only one patient (0.30%) died because of the infection, and 47.43% of them were cured. These data are similar to those of previous studies, which identified that the virus infection is self-limiting^(22)^, with less than 1% lethality rate^(22-24)^.

### Study limitations

This study has some limitations, such as the fact that it is a retrospective study, which makes it impossible to collect additional data on clinical manifestations. Besides, there are still open cases, which are constantly being updated and followed up by the State Health Department.

### Contributions to the field of Nursing, Health, or Public Policies

The results of this study may contribute to health practices and, consequently, in the field of public policies, due to the greater knowledge about the disease and, thus, its management. Moreover, it is of utmost importance to raise awareness and educate the population, especially those groups classified as higher risk; thus, the aim is to prevent infection and, consequently, reduce the transmission and spread of the disease^(24)^. This work also raises the reflection on the need to evaluate the effectiveness of existing vaccines and vaccination strategies.

## CONCLUSIONS

During the study period, 759 suspected, confirmed, and probable cases of monkeypox infection were reported in the state of Minas Gerais, with 35.44% suspected, 53.75% confirmed, and 10.81% probable. Most of the confirmed cases were male at birth, with a median age of 33 years, in which individuals self-identified as cisgender, homosexual, and had multiple partners. They showed symptoms of sudden onset fever and rash, with characteristics of multiple lesions, including in the genital region. Regarding the evolution of the confirmed cases, one patient died because of the infection, and 47.43% of them were cured.

The data found in this study are important for greater knowledge about the definition of cases of the disease and means of infection control, to contribute to better management of the disease and care in health services. This is a recent outbreak, so further research is needed for a better investigation, especially because the characteristics of the current outbreak differ from those previously registered.
